# Validated Methods for Removing Select Agent Samples from Biosafety Level 3 Laboratories

**DOI:** 10.3201/eid2611.191630

**Published:** 2020-11

**Authors:** Alexandria E. Kesterson, John E. Craig, Lara J. Chuvala, Henry S. Heine

**Affiliations:** Author affiliation: University of Florida, Orlando, Florida, USA

**Keywords:** select agents, filtration, BSL-3, biosafety level 3, sterility, methanol, validation, sample removal, Francisella tularensis, Burkholderia pseudomallei, Burkholderia mallei, Yersinia pestis, Bacillus anthracis, bacteria

## Abstract

The Federal Select Agent Program dictates that all research entities in the United States must rigorously assess laboratory protocols to sterilize samples being removed from containment areas. We validated procedures using sterile filtration and methanol to remove the following select agents: *Francisella tularensis*, *Burkholderia pseudomallei*, *B. mallei*, *Yersinia pestis*, and *Bacillus anthracis*. We validated methanol treatment for *B. pseudomallei*. These validations reaffirm safety protocols that enable researchers to keep samples sufficiently intact when samples are transferred between laboratories.

The Federal Select Agent Program (FSAP), which is jointly administered by the Centers for Disease Control and Prevention and the US Department of Agriculture, designates high-risk organisms and guidelines for their safe handling. FSAP defines Tier 1 select agents as organisms that have the potential to be used as biological weapons (*1*). These organisms might infect humans, important agricultural species of plant and animal origin, or both. Various safety and security measures prevent these organisms from being inadvertently released into the environment or obtained by persons without authorized access. For example, researchers can handle these organisms only within Biosafety Level (BSL) 3 or -4 laboratories. Transfer of these agents into, out of, or between laboratories must be well-documented to ensure the safety of the public and research personnel (*1*, 2). In 2015, failures in the sample removal protocols led the US Army to inadvertently ship live *Bacillus anthracis* spores to several laboratories in the United States and other countries (*3*). *B. anthracis* is a Tier 1 select agent and therefore subject to the rules of FSAP. These samples were thought to have been inactivated by radiation, but lapses in protocol resulted in incomplete sterility (*3*). Afterward, the FSAP created additional regulations and guidance on how samples potentially containing select agents could be removed from BSL-3 and -4 laboratories.

FSAP requires that each inactivation or sterility method for sample removal be individually tested and validated, ensuring that these methods account for assay variability and technical limits of detection (*2*). The new guidance requires the entity developing the procedure to assess the risk that live material will remain in an inactivated sample (*2*). The FSAP recognizes that checking the sterility of all samples is impossible, but laboratories should minimize the risk for a viable select agent remaining within a sample believed to be inactivated. In addition, if an entity changes an already validated procedure, the entity must revalidate that procedure (*2*). Entities might develop their procedures from commonly accepted practices or from methods described in the literature (*2*). The entity must then use the appropriate controls to validate the effectiveness of the procedure. We defined the term validate to mean that a protocol, if followed exactly, renders select agent–containing samples sterile at the bacterial concentrations stated and that the sterility verification procedures identify protocol failures.

Our laboratory at the University of Florida (Orlando, FL, USA) evaluates therapeutics for the Tier 1 select agents *Francisella tularensis*, *Burkholderia pseudomallei*, *B. mallei*, *Yersinia pestis*, and *Bacillus anthracis*. We frequently conduct studies in which serum, plasma, bronchoalveolar lavage (BAL) fluid, or spent media must be transferred from the BSL-3 to the BSL-2 laboratory to conduct specific assays. These samples must be sufficiently intact so that we can evaluate drug, cytokine, chemokine, or enzyme levels and other host or bacterial components of interest. In most instances, chemical inactivation of the samples is not advisable. We selected 0.2-μm centrifuge filtration as the most effective method to sterilize small volumes of select agent–containing samples while maintaining other components in the samples. We describe and validate a standardized method using several different matrices.

Measuring the intracellular levels of antimicrobial drugs in the BAL fluid is sometimes necessary to determine the amount of compound penetrating the site of infection within the cell. When it is necessary to measure the intracellular concentration, we treat the BAL cell pellet and the BAL fluid as 2 independent samples. Because the cell pellet sample cannot be filtered, we describe an additional procedure for removing BAL cell pellets from the containment laboratory.

## Materials and Methods

### Biosafety

We tested all protocols in a BSL-3 laboratory at the University of Florida, which is registered and licensed with the Centers for Disease Control and Prevention and the Animal and Plant Health Inspection Service, US Department of Agriculture, to conduct select agent research. The containment laboratory uses a high-efficiency particulate air filter to decontaminate discharged air. All staff must don facility-dedicated scrubs, Tyvek suits (Dupont, https://www.dupont.com), respiratory protection, double gloves, and shoe covers. All bacterial work is performed in a class II Biosafety cabinet, and all waste is removed using pass-through autoclaves.

### Bacterial Strains and Growth Conditions

We used the following strains from the Biodefense and Emerging Infections Resource Repository: *B. anthracis* (Ames), *Y. pestis* (CO92), *F. tularensis* (SchuS4), *B. pseudomallei* (1026b), and *B. mallei* (China 7). We isolated *B. anthracis* spores according to Leighton and Doi (*4*) and maintained the spores in refrigerated sterile water at »1 × 10^10^ CFU/mL. We verified this concentration by serial dilution in sterile water onto sheep blood agar plates as previously stated (*5*).

We cultured *Y. pestis* CO92 from frozen stock on sheep blood agar (Becton Dickinson, https://www.bd.com) and incubated it for 48 h at 28°C. We then removed colonies from the stock plate and suspended them in 1 mL heart infusion broth (Becton Dickinson). We added this suspension to 100 mL heart infusion broth containing 2 mL 10% xylose (Indofine, https://indofinechemical.com). We incubated this mixture in a 500 mL flask with agitation for 18–24 h.

We then cultured *B. mallei* China 7 and *B. pseudomallei* 1026b from frozen stock vials on tryptic soy agar and incubated them at 35°C for 24–48 h to generate a stock plate of each strain. We selected 2–3 colonies from each incubated stock plate and inoculated them in brain heart infusion (BHI) broth (Becton Dickinson) overnight culture. We then incubated the cultures at 35°C with agitation for 16–20 h.

We also cultured *F. tularensis* SchuS4 from frozen stock onto chocolate agar (Becton Dickinson) and incubated it at 35°C for 48 h. We selected colonies from the agar plate and used them to inoculate a BHI culture containing 2% Isovitalex (Becton Dickinson). We incubated this culture for 18–20 h at 35°C with agitation.

### Matrices

We tested the filtration protocol with murine lung BAL fluid, serum, plasma, and the listed culture mediums (Table 2). For the spore preparation, we used BHI as the culture media. We purchased the murine serum, plasma, and BAL from BioreclamationIVT (https://bioivt.com). We used mouse plasma from Balb/c mice collected in sodium citrate–containing tubes and pooled across sex. We also used mouse BAL and serum from Balb/c mice and pooled across sex.

**Table 2:** Preparation of select agents in different matrices*

Agent	CFU/mL (matrix)	BAL fluid	Serum and plasma, µL	Culture	BAL cell pellet
*Bacillus anthracis*	10^10^ (spore prep†)	20 μL	20§	20 µL	NT
*Yersinia pestis*	10^9^ (overnight culture)	Resuspend pellet¶	20	Resuspend pellet¶	NT
*Burkholderia mallei*	10^9^ (overnight culture)	Resuspend pellet#	20**	Resuspend pellet††	NT
*Burkholderia pseudomallei*	10^9^ (overnight culture)	200 μL + 1.8 mL BAL	20‡‡	Resuspend pellet§§	2 × 10^6^ CFU
*Francisella tularensis*	10^9^ (overnight culture‡)	20 μL	20¶¶	Resuspend pellet##	NT

*BAL, bronchoalveolar lavage; NT, not tested. †Spores for aerosol challenge were maintained in sterile water and diluted to the nebulizer-challenge concentration of »1 × 10^10^ CFU/mL. ‡All broth cultures will require a 2% supplement with Isovitalex (Becton Dickinson, https://www.bd.com) to obtain growth of *F*. *tularensis*. §Dilute spore prep 1:1000; transfer 20 μL to serum and plasma. ¶Centrifuge 20 mL of overnight culture, resuspend pellet in 2 mL BAL fluid or culture media. #Centrifuge 2 mL of overnight culture, resuspend in 2 mL BAL fluid. **Dilute overnight culture 1:100; transfer 20 μL to BAL fluid. ††Centrifuge 20 mL of overnight culture, resuspend pellet in 2 mL culture media. ‡‡Dilute overnight culture 1:10 transfer 20 μL to serum or plasma. §§Centrifuge 20 mL of overnight culture, resuspend pellet in 2 mL culture media. ¶¶Dilute overnight culture 1:10 transfer 20 μL to BAL fluid. ##Centrifuge 20 mL of overnight culture, resuspend pellet in 2 mL culture media.

### Test Sample Preparation

All matrices had a final volume of 2 mL. We selected test sample starting concentrations that exceeded the maximum published bacterial concentrations (Table 1). We established a conversion factor for each species on the basis of serial dilution plate counts and optical density (OD) measurements at 600 nm (H. Heine, unpub. data). We used these conversion factors to determine the concentrations of overnight cultures and spore preparations. *Y. pestis* had a conversion factor of 5.34 × 10^8^ CFU/OD, *B. mallei* and *B. pseudomallei* 1.57 × 10^9^ CFU/OD, and *F. tularensis* 3.89 × 10^10^ CFU/OD.

**Table 1:** Maximum bacterial concentrations of select agents in tissues of infected mice*

Agent (reference)	Source of samples, bacterial load			
	Lung, per g	Cell pellet, per mL BAL	Blood, per mL	Overnight culture, per mL
*Bacillus anthracis* (*5*,*6*)	<10^8^	Not tested	<10^4^	10^8^
*Yersinia pestis* (*7*)	<10^10^	Not tested	<10^6^	10^9^
*Burkholderia mallei* (*8*–*11*)	<10^9^†	Not tested	<10^4^	10^9^
*Burkholderia pesudomallei* (*11*,*12*)	<10^8^	10^5^‡	<10^5^	10^9^
*Francisella tularensis* (*13*)	10^7^	Not tested	<10^5^	10^9^

*BAL, bronchoalveolar lavage. †References (*7*) and (*8*) use a different strain of *B. mallei*  ‡Value determined through in-house testing of lung samples.

For *B. anthracis* Ames strain, we prepared spores and spiked the different matrices. We used 20 μL of the spore preparation for BAL and culture medium samples. We diluted the spore preparation 1:1000 and used 20 μL of the diluted solution to spike each serum and plasma sample (Table 2).

We prepared test samples for *Y. pestis* from the incubated 100 mL broth culture. We took an OD reading from serially diluted broth culture and conversion factors to determine the culture concentration. We centrifuged 20 mL of this culture at 3,500 × *g* for 15 min. We then resuspended this pellet in 2 mL of BAL fluid (Table 2). We repeated the process for the culture medium. We inoculated serum and plasma samples with a uncentrifuged overnight culture (Table 2).

We prepared *B. mallei* test samples from the overnight broth cultures incubated previously. We prepared BAL fluid test samples by centrifuging 2 mL overnight broth culture at 3,500 rpm for 15 min and then resuspending the pellet in 2 mL BAL fluid. We inoculated serum and plasma with an overnight culture that had been diluted 1:100, then added 20 μL to each matrix (Table 2). We inoculated culture medium by centrifuging 20 mL of the overnight culture then suspending the pellet in 2 mL of culture media (Table 2).

We prepared *B. pseudomallei* test samples for culture medium as stated for *B. mallei* and *Y. pestis* using the conversion factor. We prepared BAL fluid samples by adding 200 μL overnight culture to 1.8 mL BAL fluid (Table 2). We inoculated serum and plasma with 20 μL of overnight culture that was first diluted 1:10 (Table 2).

We prepared *F. tularensis* samples for culture medium with a final concentration of 2% Isovitalex. We took an OD reading and used the conversion factor to concentrate samples appropriately. We centrifuged 20 mL of an overnight culture and resuspended it in culture medium with 2% Isovitalex. We spiked serum and plasma samples with 20 μL of an overnight culture that was first diluted 1:10 and inoculated BAL fluid with 20 μL of an overnight culture (Table 2). 

### Methanol Test Sample Preparation

Test samples, positive controls, and the negative control of BAL fluid for the methanol treatment procedure all had a final volume of 500 μL. We used stock plates to grow bacteria, then selected colonies and suspended them in 3 mL of sterile water for injection (GE Healthcare, https://www.gehealthcare.com). We took an OD reading at 600 nm on a spectrophotometer (ThermoFisher Scientific, https://www.thermofisher.com) using a 1 cm^2^ cuvette (ThermoFisher Scientific). We converted this value to an approximate CFU per milliliter value using a conversion factor as stated in test sample preparation. We calculated the total volume needed to spike each sample so that each sample would have 2 × 10^6^ CFU (Table 2).

### Filtration Procedure

We conducted all filtration test procedures in triplicate for each matrix type. For negative controls, we used uninoculated matrix samples. For positive controls, we used 100 μL of unfiltered inoculated test samples suspended in broth culture medium. We then placed 450 μL of each test sample into a clean 0.2 μm PALL Nanosep Bio-Inert centrifuge filter (Pall Corporation, https://www.pall.com) with a sterile microcentrifuge tube. In accordance with the manufacturer’s recommendations, we centrifuged the filters for 3 min at 14,000 × *g*. We then transferred the filtrate to a clean tube and sealed it to prevent secondary contamination. We emphasize that the filtrate collection tubes should not be sealed with the same cap used to close the centrifuge filter before spinning because this cap could be contaminated with residual unfiltered sample and thus might yield false positive outcomes. We then suspended the filtrate in 4.5 mL BHI and incubated it at 35°C for 2 d. We incubated the positive controls in the same manner. After 48 h, we checked the tubes for turbidity and plated 5 × 200 μL samples onto the appropriate media. We incubated these samples at 35°C for an additional 7 d to ensure complete sterility. We considered this method to be validated only if all 3 replicates of all matrices were sterile in both broth and agar medium. Any failure, defined here as positive growth on agar or in broth media, prompted a review of the procedures. Once we determined the cause of the failure, we made the appropriate adjustments and reconducted the procedure in 3 replicates.

### Methanol Procedure

We centrifuged BAL fluid for 5 min at 5,000 × *g*. We removed the supernatant and decontaminated it using the filtration procedure detailed in the previous section. We suspended the pellet in 500 μL of 80% methanol (ThermoFisher Scientific) and incubated it for 10 min. We placed 10% of this sample into 9.5 mL Dey-Engley neutralization broth (D/E media) (Becton Dickinson) and incubated it at 35°C for 5 d. After 5 d, we plated 200 μL of the D/E media onto 5 agar plates specific to each bacterial species and incubated them at 35°C for an additional 2 d.

For positive controls, we used D/E media inoculated with bacteria and D/E media with 80% methanol added to the same volume as the test sample (50 μL of 80% methanol into 9.5 mL D/E media). We incubated this tube for 10 min and then inoculated it with bacteria. We also used growth media specific to each bacterial species as positive controls. For negative controls, we used uninoculated D/E media and D/E media inoculated with methanol treated bacteria.

## Results

After following the described procedures, we observed that all samples (except 1) were sterilized in broth culture after 48 h incubation. The samples remained sterile after plating on agar medium incubated for 7 d (Table 3). We determined that the test sample that had not been sterilized had sustained secondary contamination from the centrifuge filter unit cap. The PALL centrifuge filters are supplied as a filter and tube unit; they do not come with sterile secondary caps. To avoid secondary contamination, we transferred the filtrate to a clean tube immediately after spinning. We also observed that all samples were sterilized after treatment with 80% methanol and after incubation in broth culture for 5 d. The samples remained sterile on agar after an additional 2 d incubation.

**Table 3:** Sterility of select agent samples after sterile filtration and methanol procedure*‡

Agent (reference)	Positive serum	Positive plasma	Positive BAL	Positive overnight culture	Positive BAL cell pellet
*Bacillus anthracis*	0/3	0/3	0/3	0/3	NT
*Yersinia pestis*	0/3	0/3	0/3	0/3	NT
*Burkholderia mallei*	0/3	0/3	0/3	0/3	NT
*Burkholderia pesudomallei*	0/3	0/3	0/3	0/3	0/3
*Francisella tularensis* (*14*)	0/6	0/6	1/6†	1/6†	NT

*BAL, bronchoalveolar lavage; NT, not tested. †Negative result caused by contaminated tube cap. ‡Total success rate for filtration: 97%

## Discussion

Validating sterility procedures is a time-intensive and costly necessity for removing select agent samples from BSL-3 laboratories. Researchers can streamline this process by publishing validated methods in peer-reviewed journals.

We described and validated reproducible procedures for select agent sample removal. However, researchers should ascertain that none of their sample is lost because of binding to the filter material. In this study, we checked 100% of the sample as a proof of concept, although we recognize the impossibility of incubating 100% of the sample to ensure sterility during actual experiments. Our laboratory now samples 10% of the filtrate to verify successful disinfection. We have found that these filters have an approximate failure rate of 0.1%; however, other researchers such as Dauphin et al. have found a failure rate closer to 3% (*14*). The differences in failure rates, variety of available filter membranes, and new methods of sterilization showcase the need for clear, detailed, and reproducible published methods.
